# Single-session photodynamic therapy combined with intravitreal bevacizumab and triamcinolone for neovascular age-related macular degeneration

**DOI:** 10.1186/1471-2415-7-10

**Published:** 2007-06-07

**Authors:** Hamid Ahmadieh, Ramin Taei, Masoud Soheilian, Mohammad Riazi-Esfahani, Reza Karkhaneh, Alireza Lashay, Mohsen Azarmina, Mohammad Hossein Dehghan, Siamak Moradian

**Affiliations:** 1 Ophthalmic Research Center, Shaheed Beheshti Medical University, Labbafinejad Medical Center, Tehran, Iran; 2 Farabi Eye Hospital, Tehran, Iran; 3 Noor Eye Clinic, Tehran, Iran

## Abstract

**Background:**

To evaluate the efficacy of triple therapy consisting of single-session photodynamic therapy (PDT), intravitreal bevacizumab (IVB) and intravitreal triamcinolone (IVT) as initial pulse therapy followed by repeat intravitreal bevacizumab injections for maintenance treatment in neovascular age-related macular degeneration (AMD).

**Methods:**

In a prospective interventional case series, patients with subfoveal choroidal neovascularization (CNV) secondary to AMD underwent pulse therapy with single-session PDT according to the standard protocol followed by 1.25 mg IVB and 2 mg IVT 48 hours later. Best corrected visual acuity (BCVA) was assessed and optical coherence tomography (OCT) and fluorescein angiography (FA) were performed prior to treatment. BCVA and OCT measurements were repeated at 6 week intervals and FA was obtained after 12 weeks and when necessary thereafter. Repeat injections of IVB were performed based on fluorescein angiographic evidence of CNV leakage.

**Results:**

This series included 17 eyes of 17 patients with mean age of 67.6 ± 7.2 years. Mean follow up duration was 50.4 ± 15.5 weeks. Mean BCVA prior to treatment was 0.74 ± 0.33 logMAR which improved to 0.52 ± 0.36 logMAR after 12 weeks (P = 0.012) and 0.41 ± 0.38 logMAR after 24 weeks (P = 0.001). Mean pretreatment central macular thickness (CMT) was 395 ± 181μ which was significantly reduced to 217 ± 69μ (P = 0.005), 231 ± 79μ (P = 0.028) and 221 ± 87μ (P = 0.05) six, twelve and twenty-four weeks after initial treatment respectively. Visual acuity improvement and CMT reduction was maintained during the follow-up period. IVB injections were repeated once, twice and three times in 10, 7 and 2 eyes at a mean interval of 20.2 ± 10.1, 19 ± 13.7 and 15 ± 1.4 weeks after initial therapy, respectively.

**Conclusion:**

Initial pulse triple therapy consisting of single-session PDT combined with IVB and IVT improves vision and reduces CMT in neovascular AMD. Repeat IVB injections maintain the visual gain from the initial combination therapy.

## Background

Photodynamic therapy is an FDA-approved treatment for neovascular age-related macular degeneration (AMD); however this treatment modality seems to stabilize, rather than improve vision [1-3]. Furthermore, need for numerous retreatments with possible adverse effects on physiological choroid [4-7] and concerns about cost are important drawbacks of monotherapy with PDT. Triamcinolone is an angiostatic steroid with inhibitory effect on choroidal microvascular endothelial cell migration and tube formation [8]. Intravitreal triamcinolone monotherapy has been used for treatment of neovascular AMD with modest short-term effects; the long-term results of these studies however have been disappointing [9-11]. Potential complications of intravitreal triamcinolone include glaucoma and cataract formation or progression. The risk of these complications increase with repeat injections [11,12]. Recent studies have shown that combining intravitreal triamcinolone with PDT is superior to monotherapy in terms of visual improvement and reduced need for retreatments [13-18]. Bevacizumab is a full-length humanized anti-VEGF antibody which has recently gained popularity for treatment of neovascular AMD. Intravitreal injection of bevacizumab may improve visual acuity, decrease retinal thickness and reduce angiographic leakage in AMD [19]. Monotherapy with bevacizumab however, necessitates multiple intravitreal injections within 4–6 week intervals and each injection carries the risk of serious complications. It has been suggested that antiangiogenic drugs may also be combined with PDT as an effective treatment modality [20-22].

Combination of PDT with intravitreal injection of an anti-angiogenesis drug and a steroid may address the multifactorial pathogenesis of AMD, improve vision and reduce the need for retreatments. Results of triple therapy with PDT, bevacizumab and dexamethasone have been reported recently [23]. We performed a pilot study to evaluate the efficacy of pulse triple therapy with single-session photodynamic therapy, intravitreal bevacizumab and intravitreal triamcinolone in all types of neovascular AMD. Repeat intravitreal injections of bevacizumab were given as maintenance therapy. Herein, we report the results of this therapeutic approach.

## Methods

The study was a prospective, interventional case series. The treatment protocol was approved by the Ethics Committee of the Ophthalmic Research Center. Patients were informed about the risks and benefits of combined therapy and written consent was obtained. Eyes with all types of active subfoveal CNV secondary to neovascular AMD with lesions less than 4 disc areas and Snellen visual acuity of 20/30 to 20/400 at baseline were included. Exclusion criteria were glaucoma, diabetic retinopathy, macular disorders other than AMD and previous treatment for CNV.

Eligible eyes were evaluated before treatment completely. Best corrected visual acuity measured by Snellen chart was recorded in logMAR. Applanation tonometry, anterior segment examination with slit lamp and fundus examination was performed. All eyes underwent color fundus photography and fluorescein angiography (FA) using a confocal scanning laser angiograph (Heidelberg Retina Angiograph II: Heidelberg engineering). Optical coherence tomography (Zeiss, Dublin, CA) was performed before intervention for all cases to measure the central macular thickness (CMT). Photodynamic therapy with verteporfin was performed according to the standard regimen. Forty eight hours after PDT, intravitreal injection of 1.25 mg/0.05 ml bevacizumab (Avastin, made for F. Hoffmann- La Roche Ltd Basel, Switzerland by Genentech, Inc., San Francisco, CA) and 2 mg/0.05 ml triamcinolone acetonide(Triamhexal, Hexal AG, Holzkirchen, Germany) was carried out at two different sites under sterile condition. A topical antibiotic, every 6 hours for 3 days, was prescribed and patients were instructed to return in case of ocular pain or redness or any deterioration of vision. Patients were examined one day and one week after injection. Topical antiglaucoma medication was prescribed if intraocular pressure (IOP) was more than 21 mmHg.

Visual acuity and optical coherence tomography (OCT) were evaluated at 6 week intervals. Fluorescein angiography was repeated at week 12 and when considered necessary thereafter. CNV activity was defined as presence of leakage from the CNV on fluorescein angiography. Eyes with active CNV underwent repeat intravitreal injection of 1.25 mg bevacizumab. Repeat PDT and intravitreal triamcinolone injections were not considered in the study protocol.

Paired T-test was used to compare findings before and after treatment.

## Results

Seventeen eyes of 17 patients (7 male and 10 female) with the mean age of 67.6 ± 7.2 years were included in this study. Lesion types were predominantly classic in 5 eyes, minimally classic in 3 eyes, occult in 8 eyes and retinal angiomatous proliferation (RAP) in one eye. Mean follow-up was 50.4 ± 15.5 weeks and all 17 patients completed the 24 week follow up (Table 1).

**Table 1:** Patient characteristics and data for each patient

**Patient no, Sex, Age (yrs)**	**CNV Type**	**Pretreatment BCVA (LogMAR)**	**24- week BCVA (LogMAR)**	**No of Retreatments**	**Follow-up (weeks)**
1, M, 75	Occult	0.60	0.60	2	61
2, F, 64	Occult	0.90	0.90	0	73
3, M, 65	Minimally classic	0.78	0.48	0	46
4, F, 54	Occult	0.48	0.00	0	24
5, M, 65	Occult	1.20	0.10	2	68
6, F, 55	Classic	0.78	0.48	2	58
7, M, 67	Classic	0.30	0.00	1	56
8, F, 60	Classic	0.78	0.00	2	59
9, F, 68	RAP	0.60	0.10	2	57
10, M, 70	Occult	0.30	0.10	3	65
11, F, 75	Occult	0.60	0.40	0	52
12, F, 74	Classic	1.20	0.40	0	25
13, M, 73	Classic	0.48	0.30	3	52
14, F, 82	Minimally classic	1.20	1.20	1	57
15, F, 67	Minimally classic	0.22	0.15	1	26
16, F, 65	Occult	1.00	1.00	0	48
17, M, 71	Occult	1.10	0.78	0	30
Mean BCVA (Snellen) ± SD		0.74 (20/100) ± 0.33	0.41 (20/50) ± 0.38		

**Figure 1:**
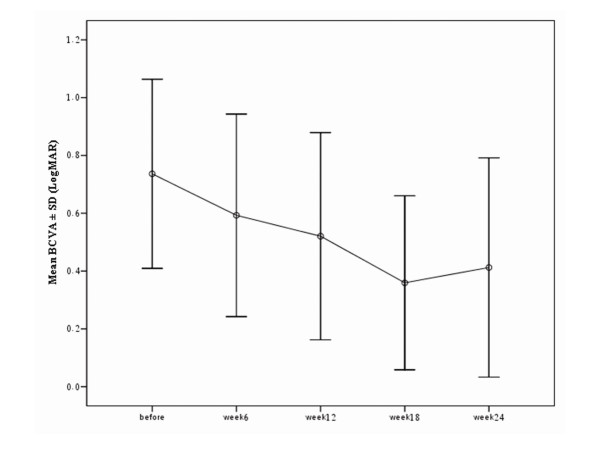
Significant visual improvement occurred after 12 weeks and persisted during the follow-up period.

**Figure 2:**
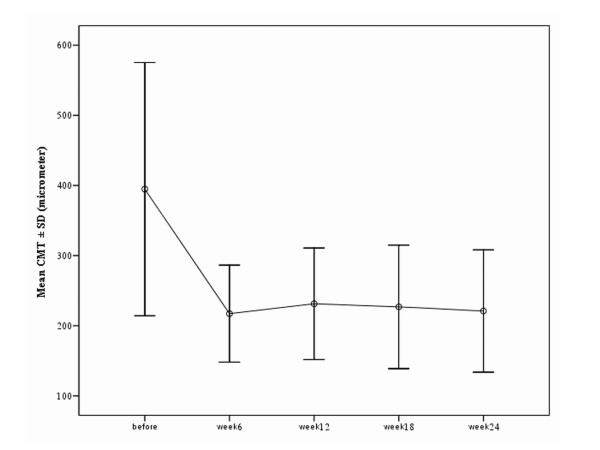
Significant reduction of CMT was observed at week 6 and continued during the follow-up.

Mean BCVA was 0.74 ± 0.33 logMAR (range: 1.20 to 0.22) before treatment (Table 1). BCVA improved to 0.52 ± 0.36 at week 12 following treatment (P = 0.012). Further improvement to 0.36 ± 0.30 logMAR (P = 0.001) and 0.41 ± 0.38 logMAR (P = 0.001) was observed at weeks 18 and 24 respectively (Figure 1). BCVA improved in 11 eyes (64.7%) and remained unchanged in 6 eyes (35.3%) 12 weeks after the initial therapy. Corresponding figures were 13 eyes (76.5%) and 4 eyes (23.5%) respectively 24 weeks after intervention (Table 1). Mean central macular thickness (CMT) was 395 ± 181μ prior to treatment. Mean CMT was reduced to 217 ± 69μ at week 6 (P = 0.005), 231 ± 79μ at week 12 (P = 0.028) and 221 ± 87μ at week 24 (P = 0.05). CMT reduction persisted during the follow-up period (Figure 2).

Seven eyes (41.2%) remained stable following the initial therapy. Follow up period for these 7 eyes ranged from 24 to 73 weeks (mean: 42.6 ± 17.6 weeks). A second intravitreal injection of bevacizumab was required in 10 eyes (58.8%), a third injection was performed in 7 eyes (41.2%) and a fourth injection was needed in 2 eyes (11.8%). Mean interval between the first and second injections and between the second and third injections were 20.2 ± 10.12 and 19 ± 13.7 weeks respectively. The interval between the third and fourth injections had a range of 14 to 16 weeks. Mean follow up for eyes with 2, 3 and 4 injections were 56 ± 11.6 weeks, 60.1 ± 5.5 weeks and 58.5 ± 9.2 weeks respectively. No adverse ocular or systemic events were observed during the follow up period.

Mean IOP was 14 ± 1.9 mmHg before treatment and increased to 17.3 ± 6.3 mmHg at week 6 and returned to pretreatment levels 12 weeks after intervention. In one patient IOP was increased to 34 mmHg after one week. Intraocular pressure was controlled in this patient with a combination of timolol eye drop bid and dorzolamide eye drop every 8 hours, which were continued for 5 weeks.

## Discussion

In this small case series, combination of single-session PDT with intravitreal bevacizumab and triamcinolone was used as pulse therapy for neovascular AMD. Our goal was to increase the efficacy of treatment and reduce side effects of monotherapy. Triple therapy resulted in significant visual improvement and CMT reduction irrespective of lesion type and also reduced the need for retreatments. Visual acuity improved significantly in the majority of cases in our series and remained unchanged in others. This treatment effect persisted during the follow up period. More than one third of our patients remained stable after the initial pulse therapy. The visual gain could be maintained in the remaining cases with few retreatments. Intravitreal bevacizumab was used for maintenance therapy to avoid the side effects of repeat PDT and repeat intravitreal triamcinolone. The average interval between the first and second treatment sessions was about 20 weeks which may reflect less need for retreatment following the initial pulse therapy.

Augustin et al recently published the results of triple therapy with PDT, bevacizumab and dexamethasone for neovascular AMD [23]. They concluded that triple therapy results in significant visual improvement after one cycle of treatment in most cases. Of 104 patients, 18 received an additional intravitreal injection of bevacizumab at a mean of 15 weeks after triple therapy. The authors employed a second cycle of triple therapy in 5 cases due to recurrent, angiographically detectable CNV activity. Avoiding repeat PDT has been one of the principles of our strategy which makes it different from the method described by Augustin et al. We limited PDT to one session in our treatment protocol for two purposes: 1- To reduce the potential side effects of PDT on the physiological choroidal vasculature, RPE and neurosensory retina; 2- To reduce the cost of treatment.

The rationale for single-session PDT emanates from the present knowledge regarding the possible adverse effects of repeat PDT on physiological choroid and even RPE and neurosensory retina [24,25]. In a study by Schmidt-Erfurth et al, the effects of multiple- PDT regimen were evaluated [5]. Persistent hypofluorescence was documented in all patients after the second and third PDT. Quantitative analysis showed 25% enlargement in the hypofluorescent area one week after the second session of PDT compared to the first [5]. In addition, multiple sessions of PDT may accelerate the risk of CNV recurrence due to aggravation of choroidal ischemia and subsequent over-expression of VEGF [5]. Augustin et al tried to reduce the potential side effects of PDT by reducing the light dose [23]. This approach is however based on a hypothesis which needs to be documented with further studies.

PDT may cause phototoxicity-induced VEGF expression and increase vascular permeability by triggering generation of free radicals and lipid peroxides [26]. Triamcinolone has been shown to suppress the early proangiogenic response in RPE cells after PDT in vitro [27]. In addition to reversing some PDT-induced adverse effects, triamcinolone may independently affect CNV [8]. There is no consensus on the optimal dose of IVT which is particularly related to concerns about adverse effects. Significant IOP elevation (ie, IOP>21 mmHg or requiring medical or surgical therapy) has been reported in 21% to 43% of eyes undergoing either 4 mg or 25 mg IVT injection [9-11]. Repeat injections of IVT are associated with more severe IOP rise [11,18]. We limited intravitreal triamcinolone to one injection and used a lower dose to reduce the adverse effects. By using the 2 mg dose of IVT, the total volume of intravitreal injections was reduced to 0.1 cc (0.05 cc IVT and 0.05 cc IVB). There was no significant difference between mean IOP before and after IVT injections in our series. Significant IOP elevation was observed only in one patient which was controlled with topical medications.

We used intravitreal bevacizumab as one of the components of pulse triple therapy. It was also used as a maintenance therapy in our series. Use of an antiangiogenic factor can counteract the PDT induced over-expression of VEGF and reduce its adverse effects. On the other hand, PDT may disrupt the architecture of CNV and help the antiangiogenic factor to affect the lesion more potently [21]. Aggio et al treated two cases of neovascular AMD with a combination of PDT and intravitreal bevacizumab. This resulted in anatomic success; however it was not equally effective in terms of VA improvement due to presence of advanced disease in both patients [20]. Dhalla et al reported the 7-month results of combined photodynamic therapy and IVB for CNV secondary to AMD [21]. Visual acuity stabilization was observed in 83% and improvement was noticed in 67%. Retreatment was needed in 37% of their cases. Costa et al performed intravitreal injection of bevacizumab one week after PDT [22]. The change in BCVA from baseline was significant at 12 weeks. Retreatment was required in 63.6% of cases at week 24 due to recurrent fluorescein leakage. Retreatments consisted of repeat PDT and intravitreal bevacizumab in these two case series.

The optimal time for intravitreal injection of bevacizumab with or without a steroid after PDT is not known. It has ranged from 16 hours[23] to 2 weeks [21] in published series. We decided to inject bevacizumab and triamcinolone 48 hours after PDT to reduce the risk of light toxicity and to counteract the adverse effects of PDT induced release of VEGF. The results of our study with this treatment strategy are encouraging however, limitations including the small number of patients and the absence of control groups make it difficult to generalize the results.

## Conclusion

Our preliminary results suggest that intravitreal bevacizumab has a synergistic effect with PDT and intravitreal triamcinolone for treatment of neovascular AMD. Using this triple therapy as an initial pulse can improve or stabilize visual acuity. In case of recurrent CNV, repeat intravitreal injection of bevacizumab may help maintain the visual gain. Further studies are required to confirm the results of this pilot study.

## Abbreviations

***LogMAR*: **Logarithm of the Minimum Angle of Resolution

***VEGF*: **Vascular Endothelial Growth Factor

***RPE*: **Retinal Pigment Epithelium

## Competing interests

The author(s) declare that they have no competing interests.

## Authors' contributions

HA participated in the design of the study, contributed in acquisition and analysis and interpretation of data, was involved in drafting the manuscript and its revising. RT participated in the design of the study, contributed in acquisition and analysis and interpretation of data, was involved in drafting the manuscript and its revising. MS participated in the design of the study, contributed in acquisition and analysis and interpretation of data, gave final approval of the version. MR participated in the design of the study, contributed in acquisition and analysis and interpretation of data, gave final approval of the version. RK participated in the design of the study, contributed in acquisition and analysis and interpretation of data, gave final approval of the version. AL participated in the design of the study, contributed in acquisition and analysis and interpretation of data, gave final approval of the version. MA participated in the design of the study, contributed in acquisition and analysis and interpretation of data, gave final approval of the version. MD participated in the design of the study, contributed in acquisition and analysis and interpretation of data, gave final approval of the version. SM participated in the design of the study, contributed in acquisition and analysis and interpretation of data, gave final approval of the version.

## Pre-publication history

The pre-publication history for this paper can be accessed here:


